# A Protocol for Low-Input RNA-Sequencing of Patients with Febrile Neutropenia Captures Relevant Immunological Information

**DOI:** 10.3390/ijms241210251

**Published:** 2023-06-16

**Authors:** Victoria Probst, Lotte Møller Smedegaard, Arman Simonyan, Yuliu Guo, Olga Østrup, Kia Hee Schultz Dungu, Nadja Hawwa Vissing, Ulrikka Nygaard, Frederik Otzen Bagger

**Affiliations:** 1 Department of Genomic Medicine, Rigshospitalet, 2100 Copenhagen, Denmark; 2 Department of Paediatrics and Adolescent Medicine, Rigshospitalet, 2100 Copenhagen, Denmark; lotte.moeller.smedegaard@regionh.dk (L.M.S.);; 3 Faculty of Health and Medical Sciences, University of Copenhagen, 2200 Copenhagen, Denmark

**Keywords:** transcriptomics, neutropenia, febrile neutropenia, low-input RNA-sequencing, paediatrics, white blood cells

## Abstract

Improved methods are needed for diagnosing infectious diseases in children with cancer. Most children have fever for other reasons than bacterial infection and are exposed to unnecessary antibiotics and hospital admission. Recent research has shown that host whole blood RNA transcriptomic signatures can distinguish bacterial infection from other causes of fever. Implementation of this method in clinics could change the diagnostic approach for children with cancer and suspected infection. However, extracting sufficient mRNA to perform transcriptome profiling by standard methods is challenging due to the patient’s low white blood cell (WBC) counts. In this prospective cohort study, we succeeded in sequencing 95% of samples from children with leukaemia and suspected infection by using a low-input protocol. This could be a solution to the issue of obtaining sufficient RNA for sequencing from patients with low white blood cell counts. Further studies are required to determine whether the captured immune gene signatures are clinically valid and thus useful to clinicians as a diagnostic tool for patients with cancer and suspected infection.

## 1. Introduction

Improved methods are needed for diagnosing infectious diseases in children with cancer. Due to the increased risk of possibly fatal infections in this population, they are hospitalised and given antibiotics when febrile [1,2]. However, the majority have fever for other reasons than bacterial infection and are exposed to unnecessary antibiotics and hospital admissions due to a lack of quick and accurate diagnostic tests [3]. This can lead to side effects and antibiotic resistance (in the society and the individual patient) [4,5,6].

Recent research has shown that host whole blood RNA transcriptomic signatures can distinguish bacterial from viral infection and inflammatory diseases [7,8,9]. This method is approaching clinical implementation and could change the diagnostic approach for children with suspected infection [10]. However, extracting sufficient mRNA from these patients to perform transcriptome profiling by standard methods is challenging due to their low white blood cell (WBC) counts. Only two studies have investigated transcriptome profiling from whole blood collected in RNA-stabilising blood tubes (PAXgene^®^) in patients with febrile neutropenia [11,12]. Wahlund et al. (2020) failed to extract sufficient RNA for sequencing in 20 out of 63 (32%) samples in children with febrile neutropenia, while Kelly et al. had to use a targeted gene panel (EdgeSeq) in 29 samples due to insufficient RNA for standard RNA-sequencing in a study of febrile neutropenia in adults. In a recent study, Haeusler et al., obtained immune gene signatures in 73 of 80 (91%) patients with febrile neutropenia using EDTA tubes [13]. The disadvantage of using EDTA tubes is the need to freeze the samples within 2 h.

Advances in single-cell mRNA-sequencing (low input protocols) have pushed the boundaries for sensitive mRNA capture and quantification and may solve the challenges in obtaining sufficient RNA from patients with low WBC [14,15,16] Flow-based protocols such as 10x Genomics can capture many single cells at the expense of gene capture [17]. Here, we hypothesise that full-transcript plate-based low-input protocol can be both sensitive enough in terms of gene capture and practical in terms of handling in the laboratory to be used for patients with low WBC counts. We previously found that the SMART-Seq^®^ HT kit protocol performed well for single-cell sequencing, with high robustness and ease of implementation [18].

In 2019, we initiated a prospective nationwide cohort study investigating unique transcriptional signatures in whole blood from children with leukaemia and suspected infection. As in previous studies, we experienced challenges detecting RNA in samples with very low WBC counts. As a result, RNA concentrations and RNA integrity numbers (RIN) were low and standard RNA-sequencing was not possible.

In this study, we aimed to compare the SMART-Seq^®^ HT kit protocol (low-input protocol) with the TruSeq Stranded RNA Library Preparation Kit (standard RNA-sequencing protocol) to investigate if the low-input protocol could recapture the same transcriptional information. We also aimed to confirm the clinical utility of the approach by applying the low-input protocol to whole blood samples from children with leukaemia and suspected infection.

## 2. Results

**Figure 1:**
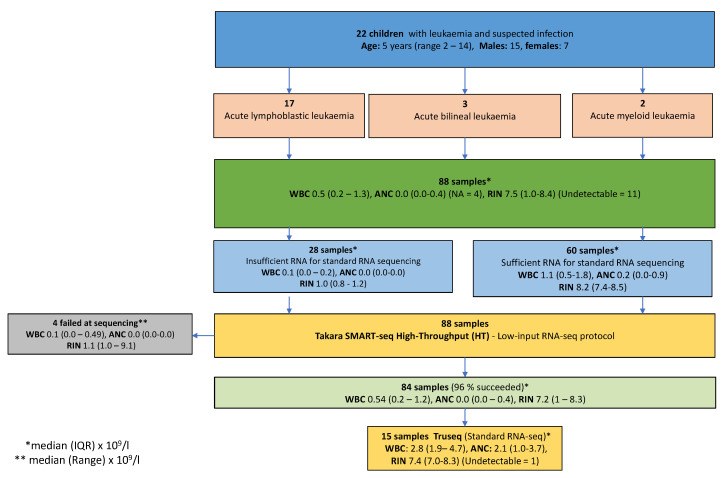
Overview of the study population sequenced either by the low-input protocol or by both low-input and standard protocols as described in the methods. A total of 88 samples from 22 patients with leukaemia and suspected infection was sequenced by the low-input protocol (Takara SMART-seq HT) (96% succeeded) and 15 of these were also processed by the standard protocol (Truseq). Samples stated by the laboratory as WBC < 0.1 × 10^9^/L and absolute neutrophil count (ANC) < 0.1 × 10^9^/L were defined as WBC = 0 and ANC = 0, respectively. * Median (IQR) × 10^9^/L, ** median (range) × 10^9^/L.

We included 22 children (15 males and 7 females) with leukaemia and a suspected infection (median age 5 years, range 2–14). Seventeen had acute lymphoblastic leukaemia, three had acute bilineal leukaemia, and two had acute myeloid leukaemia (Figure 1). From the 22 patients, 88 blood samples were analysed from 36 infection periods (median 4 samples (IQR 2–5) per patient). The samples were drawn at different time points during the infection episodes and there were no duplicates. The samples had a median WBC count of 0.5 × 10^9^/L (IQR 0.2–1.3) and an ANC of 0.0 × 10^9^/L (IQR 0.0–0.4). The median RIN was 7.5 (1.0–8.4). Eleven samples had undetectable RIN (Figure 1). In 28 of 88 (32%) samples, RNA concentration was reported as “too low” by a Qubit™ RNA High Sensitivity (HS) kit (Cat. no. Q32852). In addition, 20 of 28 samples showed “no peaks” on Bioanalyzer. The 28 samples had a median WBC count of 0.1 × 10^9^/L (IQR 0.0–0.2), including 13 children with undetectable WBC counts. The median RIN was 1.0 (IQR 0.8–1.2) (Figure 1).

We sequenced the 15 samples with both methods to test whether the low-input protocol could recapture similar transcriptional information as the standard clinical RNA-seq protocol. Furthermore, we applied the low-input protocol to 88 samples to investigate the general clinical applicability.

### 2.1. Comparing the Low-Input RNA-Seq Protocol to the Standard RNA-Seq Protocol

The 15 samples sequenced by both low-input and standard protocols had a median WBC count of 2.8 × 10^9^/L (IQR 1.9–4.7) and an ANC of 2.1 × 10^9^/L (IQR 31.0–3.7). The median RIN was 7.4 (IQR 7.0–8.3), and one had undetectable RIN) (Figure 1). The samples were selected to have enough cells and material to perform both protocols suitably. A comparison of methodological steps are in Table 1. 

### 2.2. Library Sizes and Gene Expression

**Figure 2:**
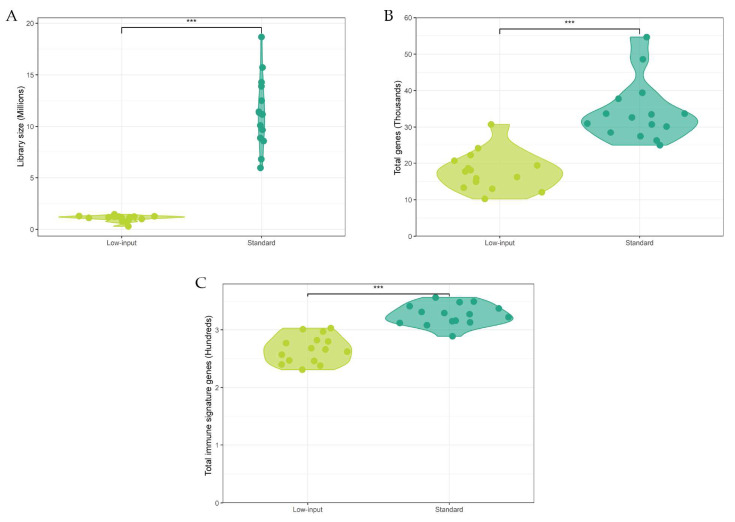
Comparative metrics from 15 samples processed with both the low-input and standard protocols. Each dot represents one sample, shaded violins represent their density distribution. ***: *p* < 0.001. (**A**) Library size (Millions) reads captured by the protocols. (**B**) Total expressed genes (thousands) captured by the protocols. (**C**) Total immune signature genes (hundreds) captured by the protocols. Immune signature genes were retrieved from the Molecular Signature Database (MSigDB).

As expected, the samples sequenced by the standard protocol had significantly larger library sizes than the low-input protocol samples (*p* < 0.001) (Figure 2A) and a correspondingly higher number of total detected genes (*p* < 0.001) (Figure 2B). The number of captured immune signature genes was also significantly higher in the standard protocol samples (*p* < 0.001) (Figure 2C). Even though the library size for the samples processed by the standard protocol on average was larger by a scale factor of 10.6×, the total number of captured genes differed only by a factor of 1.9×, and the number of immune signature genes only by a factor 1.2×. Hence, the loss of genes using the low-input protocol was smaller than the loss of sequencing reads.

**Figure 3:**
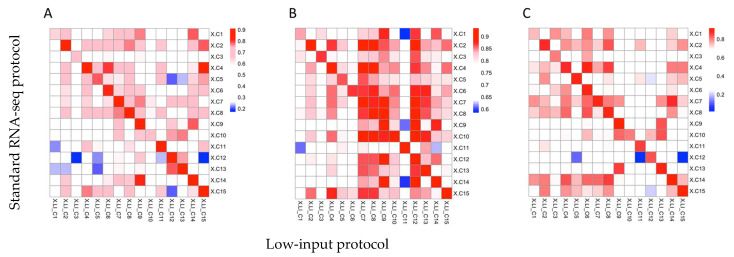
Heatmaps illustrating the correlation between gene expression levels from samples originating from the same patient analysed using a standard protocol and low-input protocol, respectively. (**A**) Union of 15 most differentially expressed genes between for each sample and the rest of the samples in the protocol. (**B**) Immune signature genes retrieved from MSigDB, and (**C**) the 38-gene signature by Herberg et al. (2016) [7]. Sample correlation is presented by the Spearman correlation coefficient and coloured according to the legend. pHeatmap (v1.0.12).

The highest inter-protocol correlation was seen between samples originating from the same patient, based on the Spearman correlation coefficient (Figure 3A–C), indicating that both methods recovered the same transcriptional signal. The correlation was most prominent in the expression of the union of the 15 most differentially expressed genes and the 38-gene signature (Figure 3A,C). As expected, the correlation of immune-related signatures showed greater inter-sample correlation because all samples were from children with suspected infection (Figure 3B).

**Figure 4:**
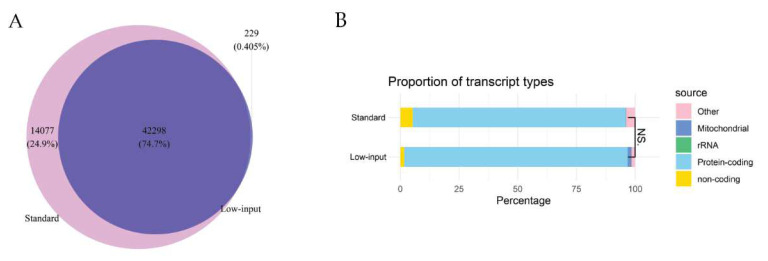
(**A**) Venn diagram illustrating the overlap of total captured genes between protocols and the genes captured uniquely in each protocol. (**B**) Bar chart visualising the proportion of different transcript types in each library. Non-coding transcript types constituted, e.g., tRNA, snRNA, snoRNA, miRNA, miscRNA, lincRNA. N.S is not significant at cut-off alpha 0.05.

Between the two protocols, 75% of transcripts were overlapping (Figure 4A), and no difference was found in the proportion of transcript types (Figure 4B).

### 2.3. Application of the Low-Input Protocol to Samples with Low White Blood Cell Counts

**Figure 5:**
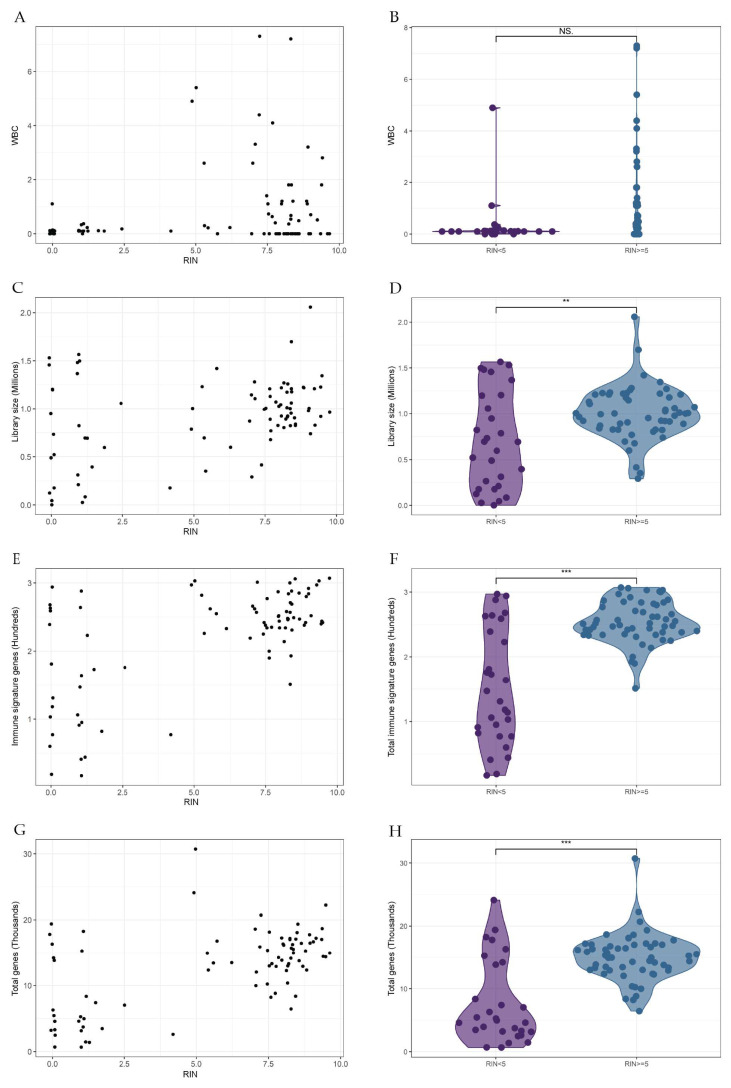
Comparative metrics from patients processed with the low-input protocol. Samples were grouped according to RIN ≥ 5 and RIN < 5. Each dot represents one sample. Significance level cut-off: NS.: *p* > 0.05, **: *p* < 0.01, ***: *p* < 0.001, (**A**,**B**) Total WBC count (×10^9^/L). (**C**,**D**) Library size (Millions), reads captured in each sample, (**E**,**F**) Immune signature genes (hundreds) retrieved from MSigDB, (**G**,**H**) total genes (thousands).

Eighty-four of 88 (95%) samples sequenced with the low-input protocol succeeded. The four samples that failed had an ANC of 0.0. Three had RIN < 5 and RNA annotated as “too low” on HS Qubit. The last sample had a RIN of 9.1 with an RNA concentration of 6.62 ng/μL measured on Nanodrop. Thus, 25 of the 28 (89%) samples that initially had insufficient RNA for sequencing succeeded with the low-input protocol. We found a wide variation of RIN among the entire study population, but no significant difference was found in the WBC count between samples with RIN ≥ 5 and RIN < 5 (Figure 5A,B).

### 2.4. Library Sizes and Gene Expression

We found a significant difference in library size between samples with RIN < 5 compared and samples with RIN ≥ 5 (Figure 5C,D). On average, there were 254 immune signature genes detected in all 84 samples, although samples with RIN ≥ 5 (sd = 0.32) had a higher mean expression (*p* < 0.001). A larger variation was observed in the number of immune genes in samples with RIN < 5 (sd = 0.90), but several samples showed comparable numbers of immune signature genes to those of RIN ≥ 5 (Figure 5E,F). The lower proportion of detected immune genes correlated with the lower number of total genes detected in samples with RIN < 5 (Figure 5G,H).

**Figure 6:**
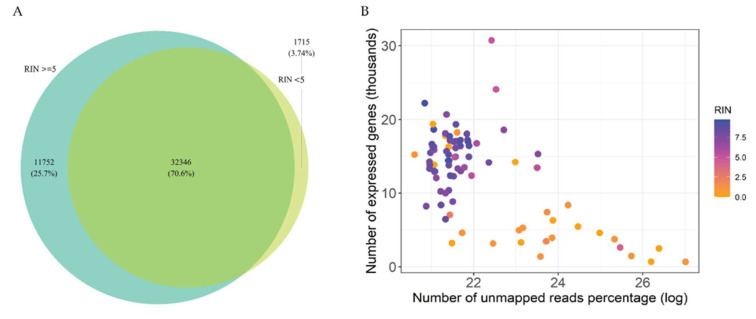
(**A**) Venn diagram illustrating the overlap of total captured genes between samples grouped according to RIN ≥ 5 and RIN < 5, as well as the genes captured uniquely in each protocol. (**B**) Dot plot visualising the relationship in the number of expressed genes (thousands) vs. number of unmapped reads (percentage) (log-scale) per sample. Each dot corresponds to one sample. Samples are coloured according to their RIN value as presented in the legend.

**Figure 7:**
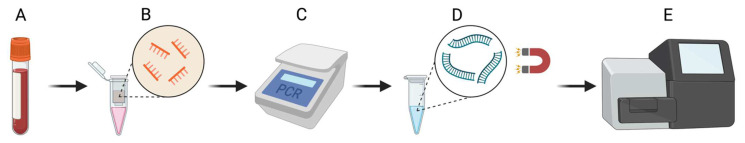
Workflow of the low-input protocol. (**A**) Patient blood samples were retrieved in PAXgene tubes and (**B**) RNA was automatically purified by QiaQube. (**C**) Reverse transcription (RT) and cDNA amplification were performed in a single step using SMART-Seq^®^ HT kit, minimising sample handling and hands-on time. (**D**) Following amplification, the samples were cleaned up and prepared for sequencing using a Nextera XT DNA library preparation kit and sequenced on (**E**) Miseq Benchtop Sequencer (Illumina, San Diego, California, USA). Illustrated using Biorender (https://biorender.com/ accessed on 1 June 2023).

A higher number of total genes (*p* < 0.001) was captured in samples with RIN ≥ 5 (sd = 3.80) compared to samples with RIN < 5 (sd = 6.65) (Figure 6G,H). Yet, 70.6% of transcripts overlapped between the two groups (Figure 7A). Furthermore, samples with RIN < 5 had a lower number of expressed genes and a higher number of unmapped genes than those with RIN ≥ 5 (Figure 6B).

## 3. Discussion

In this prospective cohort study, we succeeded in sequencing 95% of samples from children with leukaemia and suspected infection. Equivalent to the findings by Wahlund et al. (2020) [11], we found insufficient RNA for standard RNA-sequencing in one-third of the samples. Despite this, we succeeded in sequencing 89% of these samples using a low-input protocol. This is to our knowledge the first study to successfully perform non-targeted sequencing on such a large number of blood samples collected in PAXgene tubes from patients with low WBC counts. The recent advancement in RNA-sequencing techniques has lowered the threshold for RNA detection and given rise to numerous commercial kits. However, obtaining adequate RNA quantity and quality to undertake standard RNA-sequencing in samples with low white blood cell counts remained a challenge. In this study, we found a possible solution to that.

### 3.1. Comparing Low-Input to Standard RNA-Sequencing

We found that the low-input protocol was able to detect a similar level of immune-related genes to the standard protocol. We also saw a correlation in immune transcript signatures between paired samples analysed with both protocols. This indicates a comparable ability between the two protocols to capture the same immune genes in the same patient sample. Furthermore, the percentage of total captured transcripts overlapped by 75% and no difference in the proportion of transcript types was observed. The results indicate that the low-input protocol performs comparably to the standard protocol requiring a 1000-fold larger RNA input. Furthermore, the low-input protocol can obtain useful information from samples with very low starting material.

### 3.2. The Impact of RIN and WBC Counts on the Performance of the Low-Input Protocol

We detected immune signature genes in all 84 samples, including samples from patients with very low or undetectable RIN and WBC counts. This suggests that the low-input protocol is a feasible solution for obtaining useful biological information for diagnostic purposes from children with cancer and suspected infection.

We found that high-quality RNA, quantified by RIN, resulted in high coverage both in total and immune-specific genes, whereas low-quality RNA more often showed low coverage. However, many samples with low WBC count yielded RIN ≥ 5, suggesting that RNA quality sufficient for sequencing can be retrieved from patients with low WBC count.

In samples with RIN ≥ 5, the number of immune signature genes and total genes was higher, indicating that the performance of the low-input protocol is more stable in samples with higher RIN. Furthermore, we found a lower number of expressed genes and a higher number of unmapped genes (70–80%) in samples with RIN < 5, showing that the quality of the starting material is reflected in the sequencing output. The number of correctly mapped reads to the reference genome in a high-quality sample is 70–90%. A high percentage of unmapped reads signals potential contamination during sequencing or sample preparation (Sangiovanni et al., 2019) [19]. The quality of our sequenced samples was acceptable, with 70–80% of correctly mapped reads.

Some samples with RIN < 5 showed comparable numbers of immune signature genes to those with RIN ≥ 5. However, samples with RIN ≥ 5 had significantly higher levels and less variation. Thus, an effort should be made to improve the RIN of the samples with low quality.

The samples are expected to show similar expression across immune- and Herberg signature genes, but the great majority of the low-input sequencing samples had the highest correlation with the matched standard sequencing protocol control (Figure 3B,C).

It is likely that we could improve the RNA quality of samples by refining the handling process of the PAXgene tubes after sampling. Many samples were left at room temperature for 24–72 h before freezing according to the manufacturer’s instructions. Guidelines for blood sampling were based on blood samples from patients without immunodeficiencies and with sufficient RNA for standard sequencing. We suggest treating samples from this patient group with extra care to preserve the small amount of RNA. A study of the PAXgene blood tube’s preanalytical robustness showed significantly lower RIN in samples not being effectively inverted (8–10 times) upon sampling and left at room temperature for 24 h [20]. We cannot guarantee that all our samples were correctly inverted, and we could reduce the storage time at room temperature. According to several studies, the quantity and quality of RNA stored in frozen PAXgene Blood RNA tubes are stable for years, supporting our hypothesis that attention should be paid to the steps before freezing [21].

### 3.3. Perspectives

Host transcriptome profiling is considered a novel and promising way to support the clinical diagnosis of infectious diseases. Our study shows that it is possible to sequence whole blood samples with low WBC counts collected in PAXgene tubes using a low-input protocol. These findings are pivotal for the protocol to be used for diagnostic purposes of infectious diseases in patients with low WBC counts. Therefore, we see SMART-seq as feasible for general clinical implementation. Furthermore, the price per sample and hands-on time make it attractive compared to our standard clinical sequencing protocol (Table 1). The price could be further reduced by exploring alternative library preparation options [11].

The main limitation of the study was the variation in RNA quality in samples. The robustness of the low input protocol would be strengthened if the RNA quality in all the samples were high and consistent. A further limitation was the small number of patient samples sequenced using both methods.

The main strength is that the low input protocol can extract useful information from most patient samples, even from patients with low RNA quality, low WBC counts, or both.

## 4. Materials and Methods

We enrolled children under 17 years with leukaemia and suspected infection admitted to the paediatric oncology department at Copenhagen University Hospital, Rigshospitalet, from 1 May 2019, to 30 September 2019. The children were from a prospective nationwide cohort investigating unique transcriptional signatures in whole blood.

### 4.1. Blood Sampling Procedure and Sample Purification

Whole blood was collected into PAXgene^®^ Blood RNA Tubes (cat. no. 762165, Qiagen, Hilden, Germany), incubated at room temperature for a minimum of two hours and frozen to −20 °C within 72 h (Figure 7A). Before RNA extraction, the sample tubes were thawed at room temperature for at least two hours. Subsequently, we performed automated RNA purification by QiaSymphony (cat. no. 9001297, Qiagen, Hilden, Germany) and QiaQube (cat. no 9001293, Qiagen, Hilden, Germany) according to the manufacturers’ instructions (Figure 7B), thus including RNA from all blood cells for further analysis. Purified RNA was evaporated to 3 μL total sample volume.

We measured the RNA concentration using a Nanodrop (DS-11 FX+, Thermo Fisher Scientific, Waltham, MA, USA) and Qubit™ RNA High Sensitivity (HS) kit (Cat. no. Q32852, Thermo Fisher Scientific, Waltham, MA, USA) and calculated the RNA integrity number (RIN) by a Bioanalyzer 2100 instrument (G2939BA, Agilent Technologies, Santa Clara, CA, USA).

### 4.2. Low-Input Protocol Procedure

All samples were processed using a full-length transcript RNA-sequencing method Takara SMART-seq (Switching Mechanism At the 5′ end of RNA Template) High-Throughput (HT) kit (cat nr. 634862, Takara Bio Inc., Kusatsu, Shiga, Japan) (hereafter termed “low-input protocol”) in combination with Nextera XT DNA library preparation kit (cat nr. FC-131-1096, Illumina, San Diego, CA, USA) according to recommendations from the manufacturers (Figure 7C,D).

### 4.3. Library Preparation

We sorted 3 μL purified RNA samples into a 9.5 μL FACS dispensing solution consisting of 0.95 μL 10× Lysis buffer, 0.05 μL RNase inhibitor and 1 μL 3′ SMART-Seq CDS primer II A, 7.5 μL nuclease-free H_2_O. Subsequently, 20 cycles of PCR amplification were applied as recommended by the manufacturer (Figure 7C,D).

### 4.4. Sequencing

Each single cell library was diluted to a concentration of 4 nM in EB buffer + 0.1%. Between 20 and 3 μL of each 4 nM library was pooled in an Eppendorf tube. To denature the double-stranded cDNA, we mixed 5 μL of the 4 nM pool with 5 μL 0.2 nM NaOH and incubated for 5 min at room temperature. Next, the denatured sample pool was diluted to a concentration of 20 pM by mixing 10 μL 2 nM of the sample pool with 990 μL cold hybridization buffer 1 (HT1). Finally, we diluted the 20 pM sample pool to a concentration of 10 pM by mixing 500 μL of the 20 pM sample pool with 500 μL cold HT1. Samples were sequenced in groups on a Miseq benchtop sequencer (Illumina, San Diego, CA, USA) using a MiSeq reagent kit v2 300 cycles (cat nr. MS-102-2002, Illumina, USA) (Figure 7E).

### 4.5. Alignment/Trimming

Illumina sequencing raw reads were exported as fastq files and processed on a bash shell. We performed two rounds of trimming using TrimGalore! [22]. The first trimming removed Nextera XT adaptors (“CTGTCTCTTATACACATCT”), and the second trimming removed cDNA amplification adaptors (“AAGCAGTGGTATCAACGCAGAGT”). Two rounds of FastQC were performed after each trim for quality assessment of the sequencing output [23]. We used “Spliced Transcripts Alignment to a Reference” (STAR) to the Genome Reference Consortium Human Build 38 (GRCh38) to align the trimmed sequences [24]. The STAR output file comprised reads per gene for each sample. The files were merged into a single tab-delimited file. Samples that failed at sequencing quality control were discarded from further analysis.

### 4.6. Standard RNA-Sequencing Protocol

Standard RNA-sequencing was performed using a TruSeq Stranded RNA Library Preparation Kit (cat no. 20020597, Illumina, San Diego, CA, USA) (hereafter termed “standard protocol”) according to the manufacturer’s recommendations with a total purified RNA input of 0.1–1 μg.

### 4.7. Data Visualisation and Statistics

RNA-seq data were imported into an R studio as an expression matrix. We transformed the count matrix into a Single Cell Experiment object (SCE-object) using R studios (v3.6.1 Open Source, https://rstudio.com/ (accessed on 1 October 2022) package SingleCellExperiment (v1.6.0)). Data graphs were generated using ggplot2 (v3.3.0) and SinaPlot from ggforce (v0.3.3) [25]. The 15 most differentially expressed genes were between each sample and the rest of the samples from that protocol were found using DeSeq2 (v1.37.0) [26]. Heatmaps were generated using pheatmap (v1.0.12). Statistical significance was considered when *p* ≤ 0.05. Stars in plots were denoted based on a Wilcoxon test *p*-value; *: *p* ≤ 0.05, **: *p* < 0.01, ***: *p* < 0.001, ****: *p* < 0.0001. Immune signature genes were retrieved from the Molecular Signature Database, being the unique set between two signatures (MSigDB, https://www.gsea-msigdb.org/gsea/msigdb/geneset_page.jsp?geneSetName=GOBP_ACTIVATION_OF_IMMUNE_RESPONSE (accessed on 1 October 2022) and https://www.gsea-msigdb.org/gsea/msigdb/cards/GOBP_INNATE_IMMUNE_RESPONSE (accessed on 1 October 2022)), and a 38-signature from the study by Herberg et al. 2016 [7].

## 5. Conclusions

We found that a low-input protocol may be a solution to the challenge in obtaining sufficient RNA for sequencing from patients with low white blood cell counts. 

The challenge remains to investigate whether the captured immune gene signatures are clinically valid, as shown in studies of patients with normal WBC counts. It is a limitation related to febrile neutropenia that antibiotic treatment sometimes cannot be avoided; however, if this protocol can be successfully implemented, it can potentially help clinicians to diagnose patients with cancer and suspected infection more efficiently and prevent unnecessary antibiotic treatment and hospital admissions.

## Figures and Tables

**Table 1:** A comparison of methodological steps in the low input and the standard protocol.

Day	Low-Input Protocol Procedure (SMART-seq)	Day	Standard RNA-Sequencing Protocol (Truseq)
1	Purify RNA	1	Purify RNA
	One-step cDNA synthesis and amplification		RNA fragmentation
	Clean-up and validation of amplified cDNA		cDNA synthesis
	-		cDNA amplification
	-		Clean-up and validation of amplified cDNA
2	Final library preparation and sequencing	2	Final library preparation and sequencing

## Data Availability

Data have been deposited at Zenodo https://doi.org/10.5281/zenodo.7938679, accessed on 1 June 2023.
